# Adherence, Awareness, Access, and Use of Standard Diagnosis and Treatment Guideline for Malaria Case Management among Healthcare Workers in Meatu, Tanzania

**DOI:** 10.1155/2020/1918583

**Published:** 2020-02-18

**Authors:** Adela Budimu, Basiliana Emidi, Sixbert Mkumbaye, Debora C. Kajeguka

**Affiliations:** ^1^Faculty of Medicine, Kilimanjaro Christian Medical University College, Moshi, Tanzania; ^2^Meatu District Hospital, Simiyu, Tanzania; ^3^National Institute for Medical Research, Dar es Salaam, Tanzania; ^4^National Malaria Control Program (NMCP), Dodoma, Tanzania; ^5^Department of Clinical Laboratory, Kilimanjaro Christian Medical Centre, Moshi, Tanzania

## Abstract

**Background:**

Effective case management is a vital component of malaria control and elimination strategies. However, the level of adherence to the malaria diagnostic test and treatment guideline is not known, particularly at Meatu district. Therefore, this study aimed at determining the adherence, awareness, access, and use of standard diagnosis and treatment guidelines among healthcare workers in Meatu district.

**Method:**

This was a descriptive cross-sectional study, which enrolled a total of 196 healthcare workers in Meatu district. Healthcare workers were sampled purposively to reach the required sample size. A structured questionnaire was used for data collection.

**Results:**

Generally, 189 (96.4%) were aware of malaria treatment guidelines, while 148 (75.5%) had access and 98 (50.0%) used malaria treatment guidelines. One hundred and seven (54.6%) of all the healthcare workers adhered strictly to the diagnosis and national treatment guidelines. Ten (5.1%) partially adhered to the guideline when choosing antimalarials without confirmed malaria cases. Nonadherence to the prescription of recommended antimalarial drugs and laboratory confirmation was 79 (40.3%).

**Conclusion:**

Half of healthcare worker's adhere to malaria diagnostic test and treatment guidelines. Most the healthcare workers are aware of the malaria diagnostic test and treatment guidelines. Continued education and assessment of the personal attitudes towards malaria diagnostic test and treatment guidelines are recommended.

## 1. Introduction

Since 2010, the burden of malaria morbidity and mortality has been declined substantially in different parts of sub-Saharan Africa [1–5], which has been due to larger-scale control intervention programs and improved case management. In spite of malaria decline and technological advances in diagnosis, prevention, and treatment, malaria is still one of the global health problems.

Effective case management is a vital component of malaria control and elimination strategies [6]. All patients with suspected malaria should be treated based on accurate clinical assessment, confirming the diagnosis by microscopy or malaria rapid diagnostic test (mRDT) by testing blood samples before treatment with artemisinin-based combination therapy (ACT) [6]. In some parts of Tanzania, clinical diagnosis of fever is widely used by healthcare providers [7, 8]; however, there is development and spread of antimalarial drug resistance [9, 10], that may have resulted due to poor adherence to standard diagnosis and treatment guidelines [11].

Studies on adherence to malaria diagnosis and treatment guidelines among healthcare workers (HCWs) in Tanzania are limited [12]; however, one study reported on adherence to artemisinin-based combination therapies (ACTs) that relied on self-report [13]. The major challenge to the implementation of adherence to malaria diagnosis and treatment guidelines has been documented to be slow in giving back information on the new drug regime at health facilities [14], and this points to lack of awareness on the availability of diagnosis and treatment guidelines.

Despite reports of malaria decline in different parts of sub-Saharan Africa, reports indicate varying level of adherence to malaria diagnosis test and treatment guidelines [15–17], as well as lack of awareness on the availability of malaria diagnosis and treatment guidelines [16]. Furthermore, in many settings, national programs have devoted much in making sure that RDT is available in peripherals, and compliance to negative results will need to improve to prevent mismanagement of patients and overprescribing of anti-malarial drugs[18].

Tanzania Mainland's National Guidelines for the Diagnosis and Treatment of Malaria was introduced in 2006 [19] and revised in 2014. The guideline stipulated artemether-lumefantrine (AL) as the first-line treatment for uncomplicated malaria in both adults and children, with dihydroartemisinin-piperaquine (DHA PPQ) as a second-line treatment in cases of treatment failure [20]. Priorities for malaria case management are as follows: (1) to improve the quality of diagnostic and case management services, and (2) to maintain and improve supplies of the antimalarial drugs such as ACT in both the public and private sectors [21].

Therefore, this study was designed to determine adherence, awareness, access, and use of standard diagnosis and treatment guidelines among HCWs in Meatu district.

## 2. Methods

### 2.1. Study Design and Area

This was a descriptive cross-sectional study conducted from March to June 2017. The study was conducted at Meatu district. Meatu district is located in Simiyu Region, Tanzania. Meatu district is located between longitudes 34°8′ and 34.49″E and between latitudes 2°57′ and 4.9″S [22]. The region has a malaria prevalence of 13.4% among children and a fever prevalence of 21.4% [23], of which could be misdiagnosed as malaria cases. Meatu district has a total population of 299,619 people [24]. Meatu district has one district hospital, two health centers, and 45 dispensaries. The district has a total of 262 HCWs in general.

### 2.2. Study Population and Sampling Technique

The study included HCWs who manage malaria patients or capable of diagnosing and prescribing antimalarial medication in the district hospital, health center, and dispensaries. The study excluded all medical students who were interns. Purposively, a sampling technique was applied to select the HCWs who were ready to share their information.

### 2.3. Data Collection

#### 2.3.1. Questionnaire

Pretested semistructured questionnaires were administered to obtain information on HCWs socio-demographic characteristics, adherence awareness, accessibility, and usage of standard malaria diagnosis and treatment guidelines. The questionnaire was divided into three sections as follows. Section one: this part investigated the general information of the HCWs including social-demographic characteristics such as age, sex, and experience at work. Section two: this part addressed awareness of standard diagnosis and malaria treatment guidelines. Section three: This part focused on adherence to malaria diagnosis and treatment guidelines among healthcare providers.

#### 2.3.2. Pretesting of Questionnaire

To maximize validity, the questionnaire was pretested on relevant respondents before distribution. Ten HCWs filled the questionnaire as a pilot study, and in-depth cognitive interviews were carried out to examine how the HCWs understood and responded to the questions. Besides, two experts in the field of survey design approved the quality of the questionnaire. After the pretest, adjustments in phrasings were made, and an additional question was included. Nevertheless, the data of HCWs involved in the pretesting of the questionnaire were not included in the final analysis.

### 2.4. Definition

Adherence was categorized as strict, partial, and nonadherence [16]. Strict adherence was when the choice of antimalarial drug for the treatment of parasitologically confirmed malaria cases was restricted to the national guideline for malaria treatment. Partial adherence was when there was no parasitological confirmation of cases, but the choice of antimalarials matched the national guideline for malaria treatment. Nonadherence was when there was no parasitological confirmation of cases, and the choice of antimalarial medicines did not follow the national malaria treatment guidelines [16].

### 2.5. Data Analysis

Data were analyzed by using the IBM SPSS Statistics for Windows, Version 22.0 (IBM Corp, Armonk, NY, USA). Descriptive statistics were used to summarise frequencies, proportions, percentages, means and standard deviations, tables, and charts. Percentages and frequencies were used for categorical variables, and means and standard deviations were calculated for continuous variables. Association between the awareness, access, and use of guidelines and type of facility were tested using chi-square and Fisher's tests, and results were considered significant at *p* < 0.05.

### 2.6. Ethical Approval and Consent to Participate

The approval was granted from Kilimanjaro Christian Medical University College Research Ethics Review Committee. Permission to conduct the study was gained from the Simiyu district medical officer. Written informed consent was obtained from all HCWs who voluntarily agreed to take part in this study.

## 3. Results

### 3.1. Demographic Characteristics of the Study HCWs

A total of 196 HCWs were included in the study with a mean age (mean ± SD) of 35.09 ± 8.65. Most of the HCWs 84 (42.9%) were aged 21 to 30 years, and half of them were male 107 (54.6%). Lastly, 58 (29.6%) of HCWs were enrolled nurses. The demographic characteristics of the studied population are summarized in Table 1.

### 3.2. Awareness, Accessibility, and Use of National Malaria Treatment Guideline

**Figure 1:**
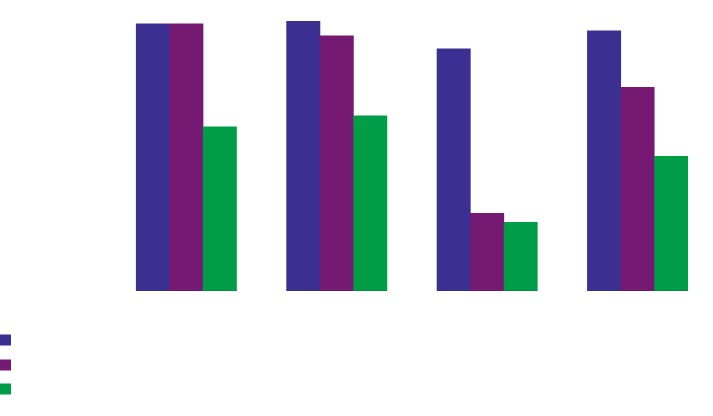
Awareness, accessibility, and use of national malaria treatment guidelines among HCWs in Meatu district.

Generally, 189 (96.4%) were aware of malaria diagnosis and treatment guidelines; additionally, of the 196 HCWs in the district hospital, health centre, and dispensary, 99 (99.0%), 37 (100%), and 53 (89.8%), respectively, were aware of malaria treatment guidelines (Fischer's exact test = 8.2, *p*=0.005). One hundred and forty eight (75.5%) had access to malaria treatment guidelines while 96 (96.0%), 35 (94.6%), and 17 (28.8%) from district hospital, health centre, and dispensary had access to malaria treatment guidelines, respectively (Fischer's Exact test = 95.58, *p* < 0.01). And lastly, 98 (50.0%) usually use malaria treatment guidelines, accounting for 59 (59.0%), 24 (64.9%), and 15 (25.4%) from district hospital, health centre, and dispensary using malaria treatment guidelines, respectively (*χ*^2^ = 20.76, *p* < 0.01) (Figure 1).

The majority of HCWs 145 (74.0%) were trained about malaria diagnosis and treatment at medical college. Half of HCWs 101 (51.5%) attended the training of malaria diagnosis and treatment. Most of study HCWs 189 (96.4%) has heard about national malaria treatment guidelines (see Table 2).

### 3.3. Adherence to Malaria Diagnosis and Treatment Guidelines

Table 3 summarizes the adherence to malaria diagnosis and treatment guidelines. Generally, 107 (54.6%) of all the HCWs adhered strictly to the diagnosis and treatment guideline, in the confirmed malaria cases, accounting for 55 (51.4%), 19 (17.8%), and 33 (30.8%) in the district hospital, health centre, and dispensary, respectively. Ten (5.1%) partially adhered to the guideline when choosing antimalarials without confirmed malaria cases. Lastly, nonadherence to the prescription of recommended antimalarial drugs and laboratory confirmation was 79 (40.3%), accounting for 43 (54.4%), 15 (19.0%), and 21 (26.6%) in the district hospital, health centre and dispensary, respectively (*χ*^2^ = 4.45, *p* > 0.05).

## 4. Discussion

Malaria continues to be one of the most devastating infectious diseases in sub-Saharan Africa. Interventions to control malaria require an integrated approach including vector control using long-lasting insecticidal nets, indoor residue sprays, provision of prompt and effective diagnosis, and treatment with effective antimalarial agents [6, 25, 26]. For this study, adherence to malaria diagnosis and treatment was measured in terms of parasitological diagnosis of malaria and treatment with the correct drug. Nonadherent treatment was defined in terms of inconsistency in the confirmatory diagnosis of malaria, prescribing of drugs other than antimalarials, and prescribing antimalarials to cases testing negative.

### 4.1. Adherence to Malaria Diagnosis and Treatment among HCWs

Poor adherence to standard diagnostic and treatment guidelines is a major cause of treatment failure and drives the emergence and spread of drug resistance [6]. WHO recommends that malaria be parasitologically confirmed by either RDT or microscopy before treatment. Following the standard guideline helps to reduce the spread of drug resistance, limit unnecessary use of antimalarial drugs, and better identify other febrile illnesses in the context of changing malaria epidemiology, and antimalarial medicines should be administered only to patients who truly have malaria [6]. In a systematic review and meta-analysis, it was found that most HCWs in sub-Saharan Africa (17% of RDT) gave negative results and patients were inappropriately prescribed with antimalarials, meaning that hundreds of thousands of patients are inappropriately diagnosed with malaria and prescribed with antimalarials [18]. Therefore, adherence to a full treatment course must be promoted.

Our study revealed that 54.6% adhere to diagnosis and treatment guidelines. These results are higher than a study done in Ogun state, Nigeria, which showed that adherence was 44.1% [16]. Moreover, our study reports nonadherence of 40.3% which is slightly higher than the study conducted in Ogun state, Nigeria, which reports nonadherence of 22.5% [16].

### 4.2. Awareness, Access, and Use of National Malaria Treatment Guidelines

Our study reports a high level of awareness of national diagnosis and treatment guidelines among HCWs at 96.4%. This is higher than findings reported by a study conducted here in Tanzania which revealed that 15.5% of HCWs were aware of the country's guidelines [27]. These results are slightly the same by a study conducted in private (94.8%) and public (98.1%) facilities [16]. Surprisingly, our study reports low proportions of HCWs who have access and use national diagnosis and treatment guidelines in dispensaries. The Tanzanian government has made it possible to establish Tanzanian health policy and has established a clear objective of achieving primary health care for all and to ensure a dispensary or health center within 5 km for everyone in the population [28]. The success of reducing the burden of infectious diseases will not be met if HCWs do not have access and use national diagnosis and treatment guidelines. Jointly to availability and use, regular training among HCWs would be important in the attainment of malaria elimination in Tanzania. Lack of access and use of national diagnosis and treatment guidelines may have contributed to nonadherence or partial adherence to the guideline. And, therefore, development and spread of ACT resistance may have higher negative influence on the recent achievements in malaria control if HCWs do not adhere to standard diagnostic and treatment guidelines. The government must ensure the availability of diagnosis and treatment guidelines to all health facilities. Also, the government should ensure job training to all HCWs to promote adherence to malaria diagnosis and treatment.

### 4.3. Strength and Limitations of the Study

The study has been able to report the level of adherence, awareness, accessibility, and use of diagnosis and treatment guidelines in Meatu district. Also, this study has some limitations. The study did not meet the required sample size due to scarcity of HCWs who meet criteria for the study; however, the present results provide a piece of baseline information on the awareness, accessibility, and use of diagnosis and treatment guidelines.

## 5. Conclusion

Generally, half of HCWs adhere to diagnosis and treatment guidelines. The majority of HCWs are aware of standard malaria diagnosis and treatment guidelines. HCWs from dispensaries have limited access and few HCWs use diagnosis and treatment guidelines in their routine clinical practices.

## Figures and Tables

**Table 1:** Demographic characteristics of the participants *N* = 196.

Variables		Frequency (*n*)	Percent (%)
*Age in years*	21–30	84	42.9
	31–40	61	31.1
	41–50	39	19.9
	51–60	12	6.1
*Sex*	Male	107	54.6
	Female	89	45.4
*Type of health facility*	District hospital	102	52
	Health center	36	18.4
	Dispensary	58	29.6
*Cadre*	Medical doctor	3	1.5
	Assistant medical officer	9	4.6
	Clinical officer	42	21.4
	Assistant clinical officer	12	6.1
	Assistant nurse officer	25	12.8
	Medical attendants	47	24
	Enrolled nurse	58	29.6
*Work experience*	<5 years	72	36.7
	5–15 years	80	40.8
	>15 years	44	22.4

**Table 2:** Awareness of national malaria treatment guidelines.

Variables	*n*	%
Training of malaria diagnosis and treatment		
At work	51	26.0
College	145	74.0
Attended regular on-job training concerning diagnosis and treatment of malaria		
No	95	48.5
Yes	101	51.5
Ever heard about national malaria treatment guidelines		
No	7	3.6
Yes	189	96.4

**Table 3:** Adherence to malaria diagnosis and treatment guidelines (*N* = 196 if no other indication is present).

Adherence	District hospital	Health center	Dispensary	Total
Strictly	55 (51.4)	19 (17.8)	33 (30.8)	107 (54.6)
Partially	2 (20.0)	3 (30.0)	5 (50.0)	10 (5.1)
Nonadhered	43 (54.4)	15 (19.0)	21 (26.6)	79 (40.3)

## Data Availability

The data used to support the findings of this study are available from the corresponding author upon request.

## Abbreviations

ACT: Artemisinin-based combination therapy
MRDT: Malaria rapid diagnostic test
NIMR: National Institute for Medical Research
SPSS: Statistical package for social science
WHO: World Health Organization.
